# Role of *Anopheles* (*Cellia*) *rufipes* (Gough, 1910) and other local anophelines in human malaria transmission in the northern savannah of Cameroon: a cross-sectional survey

**DOI:** 10.1186/s13071-016-1933-3

**Published:** 2017-01-11

**Authors:** Raymond N. Tabue, Parfait Awono-Ambene, Josiane Etang, Jean Atangana, Antonio-Nkondjio C, Jean C. Toto, Salomon Patchoke, Rose G.F. Leke, Etienne Fondjo, Abraham P. Mnzava, Tessa B. Knox, Alexis Tougordi, Martin J. Donnelly, Jude D. Bigoga

**Affiliations:** 1 Faculty of Science, Department of Biochemistry, University of Yaounde I, P.O. Box 813- Messa, Yaounde, Cameroon; 2 National Reference Unit for Vector Control, The Biotechnology Center, University of Yaoundé I, P.O. Box 3851-Messa, Yaoundé, Cameroon; 3 Ministry of Public Health, National Malaria Control Programme, P.O. Box 14386, Yaoundé, Cameroon; 4 Laboratoire de Recherche sur le Paludisme, Organisation de Coordination pour la lutte Contre les Endémies en Afrique Centrale (OCEAC), P.O. Box 288, Yaoundé, Cameroon; 5 Global Malaria Programme, World Health Organization, Avenue Appia 20, Geneva, Switzerland; 6 WHO country Office in Cameroon, PO BOX 155, Yaounde, Cameroon; 7 Malaria Programme, Wellcome Trust Sanger Institute, Hinxton, Cambridge, UK; 8 Department of Vector Biology, Liverpool School of Tropical Medicine, Pembroke Place, Liverpool, L3 5QA UK

**Keywords:** Malaria, Transmission, *Anopheles*, Local vectors, Northern Cameroon

## Abstract

**Background:**

As part of a study to determine the impact of insecticide resistance on the effectiveness of long-lasting insecticide treated nets (LLINs) in the north of Cameroon, the unexpectedly high density and anthropophilic behaviour of *Anopheles rufipes* lead us to investigate this species bionomics and role in human malaria parasite transmission.

**Methods:**

For four consecutive years (2011–2014), annual cross-sectional sampling of adult mosquitoes was conducted during the peak malaria season (September-October) in three health districts in northern Cameroon. Mosquitoes sampled by human landing catch and pyrethrum spray catch methods were morphologically identified, their ovaries dissected for parity determination and *Anopheles gambiae* siblings were identified by molecular assay. Infection with *P. falciparum* and blood meal source in residual fauna of indoor resting anopheline mosquitoes were determined by enzyme-linked-immunosorbent assays.

**Results:**

*Anopheles gambiae* (*sensu lato*) (*s.l.*) comprised 18.4% of mosquitoes collected with *An. arabiensis* representing 66.27% of the sibling species. The proportion of *An. rufipes* (2.7%) collected was high with a human-biting rate ranging between 0.441 and 11.083 bites/person/night (b/p/n) and an anthropophagic rate of 15.36%. Although overall the members of *An. gambiae* complex were responsible for most of the transmission with entomological inoculation rates (EIR) reaching 1.221 infective bites/person/night (ib/p/n), *An. arabiensis* and *An. coluzzii* were the most implicated. The roles of *An. funestus*, *An. pharoensis* and *An. paludis* were minor. *Plasmodium falciparum* circumsporozoite protein rate in *Anopheles rufipes* varied from 0.6 to 5.7% with EIR values between 0.010 and 0.481 ib/p/n.

**Conclusions:**

The study highlights the epidemiological role of *An. rufipes* alongside the members of the *An. gambiae* complex, and several other sympatric species in human malaria transmission during the wet season in northern Cameroon. For the first time in Cameroon, *An. rufipes* has been shown to be an important local malaria vector, emphasising the need to review the malaria entomological profile across the country as pre-requisite to effective vector management strategies.

## Background

The discovery at the turn of the nineteenth century that malaria was transmitted by mosquitoes initiated an outburst of interest in the description and implication of various species in malaria transmission [1]. Human plasmodial species are transmitted by mosquitoes of the genus *Anopheles*, which includes 465 formally recognised species [2]. Amongst these, over 140 species have been identified in Africa [3, 4], of which less than 20 have been shown to support the development and propagation of human *Plasmodium* species [5]. However, their relative contribution to malaria transmission greatly varies depending on their behaviour (host seeking, feeding and resting) and densities as influenced by environmental conditions. These differences in *Anopheles* behaviour and density, along with vector longevity, are key factors driving malaria transmission and epidemiological patterns observed across Africa [6, 7]. Therefore, in areas with numerous potential vectors, an in-depth understanding of vectors dynamics is fundamental to designing interventions tailored to the local eco-epidemiological situation [8, 9].

The malaria vectorial system in Cameroon is very complex. To date, over 50 *Anopheles* species have been described which are heterogeneously distributed across the eco-epidemiological zones of the country. Fifteen species have been demonstrated to support the development and spread of human *Plasmodium* spp.: *An. gambiae* Giles, 1902; *An. funestus* Giles, 1900; *An. arabiensis* Patton, 1905; *An. nili* (Theobald, 1904) and *An. moucheti* Evans, 1925 are classified as major vectors [10]. The other species amongst which are *An. pharoensis* and *An. paludis* play only a minor localised secondary role in transmission [11–14], and they may help to augment or extend the malaria transmission period [15, 16]. For the first time we report the importance of *An. rufipes* (Gough, 1910), as vector of human *Plasmodium* in Cameroon. Although a few studies have attempted to associate *An. rufipes* with human malaria transmission [17, 18], this species has generally been reported to be zoophilic [3, 4] with high densities in rice-growing areas [19, 20]. Even though this species has often been collected landing on human volunteers in several studies in Cameroon [21–23], its role in malaria transmission has never been investigated.

## Methods

### Study site and design

**Fig. 1:**
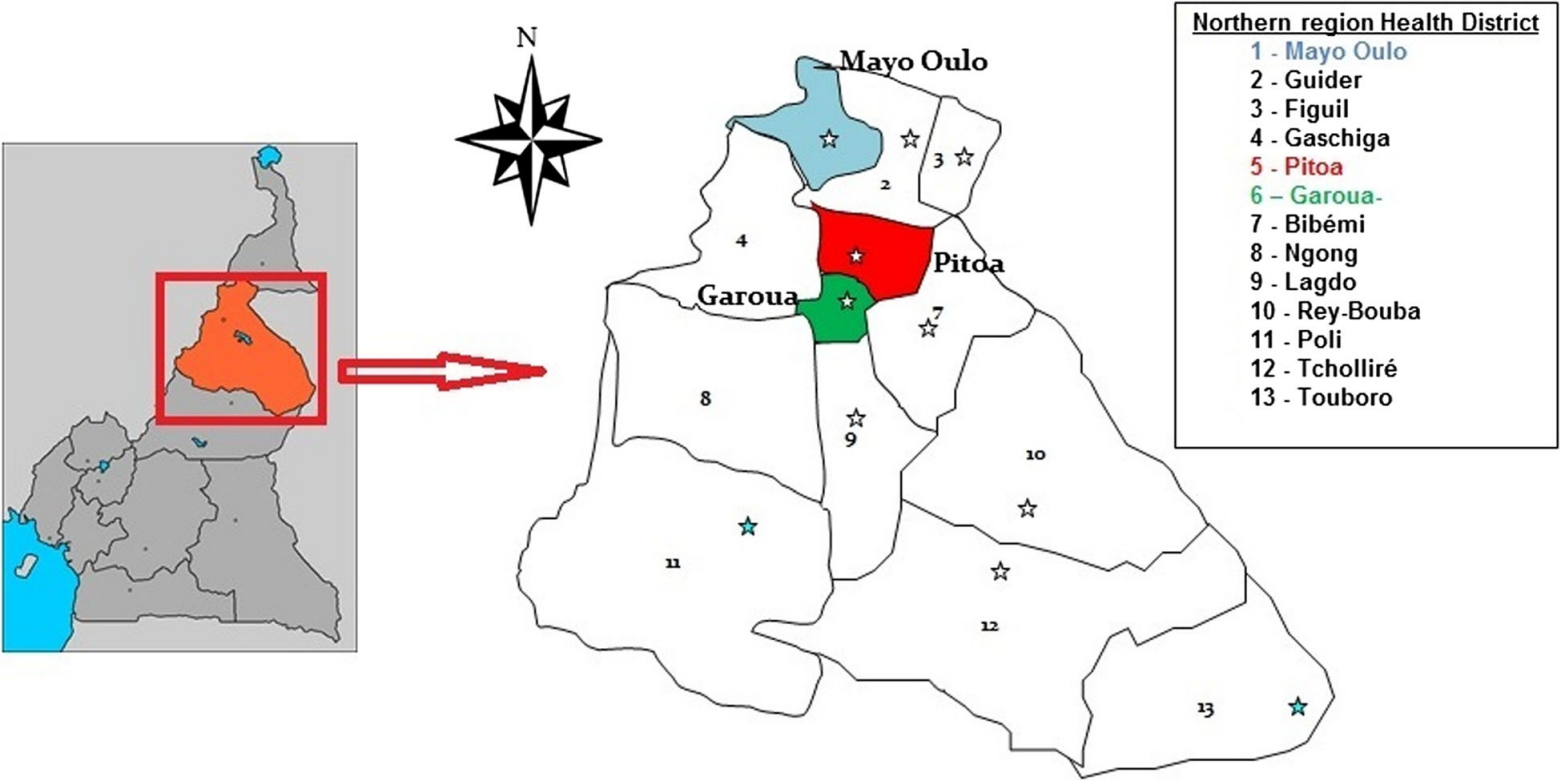
Map of the North region of Cameroon showing the study Health Districts (1, 5 and 6)

For four consecutive years (2011–2014), annual cross-sectional sampling of adult *Anopheles* mosquitoes was conducted in Northern Cameroon during the peak malaria season (September-October) in the health districts (HD) of Pitoa (9°23'0"N, 13°32'0"E), Garoua (9°18'0"N, 13°24' 0" E) and Oulo Mayo (9°7'34"N, 13°37'20"E) (Fig. 1). A total of 38 villages (12, 17 and 9 villages from each HD, respectively) were included in the assessment. Pitoa is a peri-urban area with about 108,611 inhabitants. The main economic activity is farming with major crops cultivated being rice, cotton, millet, sorghum and maize. On the other hand, Garoua is an urban area with a population of around 316,957 with rural suburbs that depend almost entirely on agriculture for subsistence. Mayo Oulo is predominantly rural, highland area with around 91,501 inhabitants. Unlike in the suburbs of Garoua where typically corn, tomatoes and eggplant are grown, in Mayo Oulo major crops cultivated are maize, beans and peanuts. All three areas have a Sahelian-type climate with an annual average rainfall of 700–1,000 mm and annual average temperature of 26–33 °C. *Anopheles gambiae* (*s.l.*) is the major malaria vector along with typical sahelian vectors including *An. funestus* and *An. pharoensis*, and *Plasmodium falciparum* is the most prevalent malaria parasite species [24, 25].

### Field sampling of adult mosquitoes

Mosquitoes were sampled using both the human landing catch (HLC) and pyrethrum spray collection (PSC) methods. Mosquito collections took place concurrently in all 38 villages within the three HDs. HLCs were performed for two consecutive nights from 18:00–06:00 h. Mosquitoes were collected indoors and outdoors in three randomly selected houses (at least 50 m apart) in each village each night with rotation between houses at different locations. A team of four trained volunteers per house (two working during the first half of the night and the others during the second half of the night) made the collection, with one sitting inside the house and the other on the veranda collected female mosquitoes as they landed on exposed lower limbs. The mosquitoes were sorted by genus and the anophelines identified morphologically using the morphological identification keys of Gillies & de Meillon [3] and Gillies & Coetzee [4]. The ovaries of a proportion of unfed mosquitoes were dissected for parity determination [26]. All dissected and those undissected mosquitoes were individually stored in tubes containing silica gel for subsequent laboratory analyses. PSCs for indoor resting mosquitoes were performed once in the morning (06:00–08:00 h) as described by Service et al. [27], and the mosquitoes were identified morphologically with their physiological status recorded. Specimens were individually stored desiccated in tubes for later laboratory analyses.

### Laboratory analysis of *Anopheles* spp. mosquitoes

A portion of specimens from each collection method belonging to the *Anopheles gambiae* complex were further identified using molecular assays. DNA from the legs and wings of each individual specimen was extracted [28] and PCR amplification was conducted to determine species [29]. The head and thorax portion of each *Anopheles* female collected was separated from the rest of the body, homogenized in grinding buffer (0.5% Casein, 0.1 N NaOH, 1 × PBS) and examined for the presence of *Plasmodium falciparum* circumsporozoite protein (CSP) by enzyme-linked-immunosorbent assay (ELISA) [30, 31]. This was to avoid cross reactivity with animal blood as reported previously [32]. To minimize false positive CSP ELISA, only high absorbance readings were considered (mean plus three standard deviations of negative controls). Blood-fed females resting indoors were screened for the source of blood meal by ELISA as described by Beier et al. [33] and modified by Lardeux et al. [34]. Monoclonal antibodies against human, cow, pig and sheep blood were used.

### Data analysis

Man biting rate (ma) was calculated as the average number of bites from *Anopheles* species received per person per night of collection. Infection Rate (IR) was calculated as the proportion of *Anopheles* species tested positive for *P. falciparum* CSP by ELISA, while the Human Blood Index (HBI) represented the proportion of *Anopheles* species identified by ELISA to have fed on human blood. The Entomological Inoculation Rate (EIR) was determined as the product of the Infection Rate (IR) and the man biting rate (ma). Data were analysed using SPSS Statistics 17.0. A Chi-square test was used to determine variable significance and the threshold for statistical significance was set at *P* < 0.05.

## Results

### Mosquito composition and anopheline density

A total of 80,689 mosquitoes were collected by HLC, comprising 61.7% *Culex* spp., 26.7% *Anopheles* spp., 11.1% *Mansonia* spp., 0.3% *Aedes* spp. and 0.2% *Coquillettidia* spp*.* Overall 21,571 *Anopheles* mosquitoes were identified morphologically (Table 1), of which there were 14,858 *An. gambiae* (*s.l.*), 2,169 *An. rufipes*, 1,802 *An. pharoensis*, 1,259 *An. funestus* and 659 *An. paludis*. The remaining 1% (824) of anopheline species were scarce and their occurrence varied greatly between the villages and health districts (Table 1).

**Table 1:** Composition and abundance of mosquitoes by species and study health district

Species	Garoua	Pitoa	Mayo Oulo	Total
	% (*n*)	% (*n*)	% (*n*)	% (*n*)
*Anopheles* spp.				
*An. gambiae* (*s.l.*)	14.7 (5,138)	22.7 (7,774)	17.0 (1,946)	18.4 (14,858)
*An. rufipes*	0.7 (234)	2.1 (722)	10.6 (1,213)	2.7 (2,169)
*An. pharoensis*	2.8 (965)	2.0 (699)	1.2 (138)	2.2 (1,802)
*An. funestus*	1.4 (496)	1.7 (591)	1.5 (172)	1.6 (1,259)
*An. paludis*	0.8 (270)	1.0 (357)	0.3 (32)	0.8 (659)
Other *Anopheles* spp.	0.1 (29)	1.4 (479)	2.8 (316)	1.0 (824)
Total 1	20.4 (7,132)	31.0 (10,622)	33.4 (3,817)	26.7 (21,571)
Culicines				
*Culex* spp.	74.5 (26,111)	50.0 (17,111)	57.3 (6,554)	61.7 (49,776)
*Mansonia* spp.	4.6 (1,618)	18.8 (6,434)	7.8 (886)	11.1 (8,938)
*Aedes* spp.	0.1 (20)	0.1 (49)	1.5 (171)	0.3 (240)
*Coquillettidia* spp.	0.4 (156)	0.0 (7)	0.0 (1)	0.2 (164)
Total 2	79.6 (27,905)	69.0 (23,601)	66.6 (7,612)	73.3 (59,118)
Total 1 + 2	100 (35,037)	100 (34,223)	100 (11,429)	100 (80,689)

*Abbreviation*: *n* number collected

### Molecular identification of *Anopheles gambiae* siblings

**Fig. 2:**
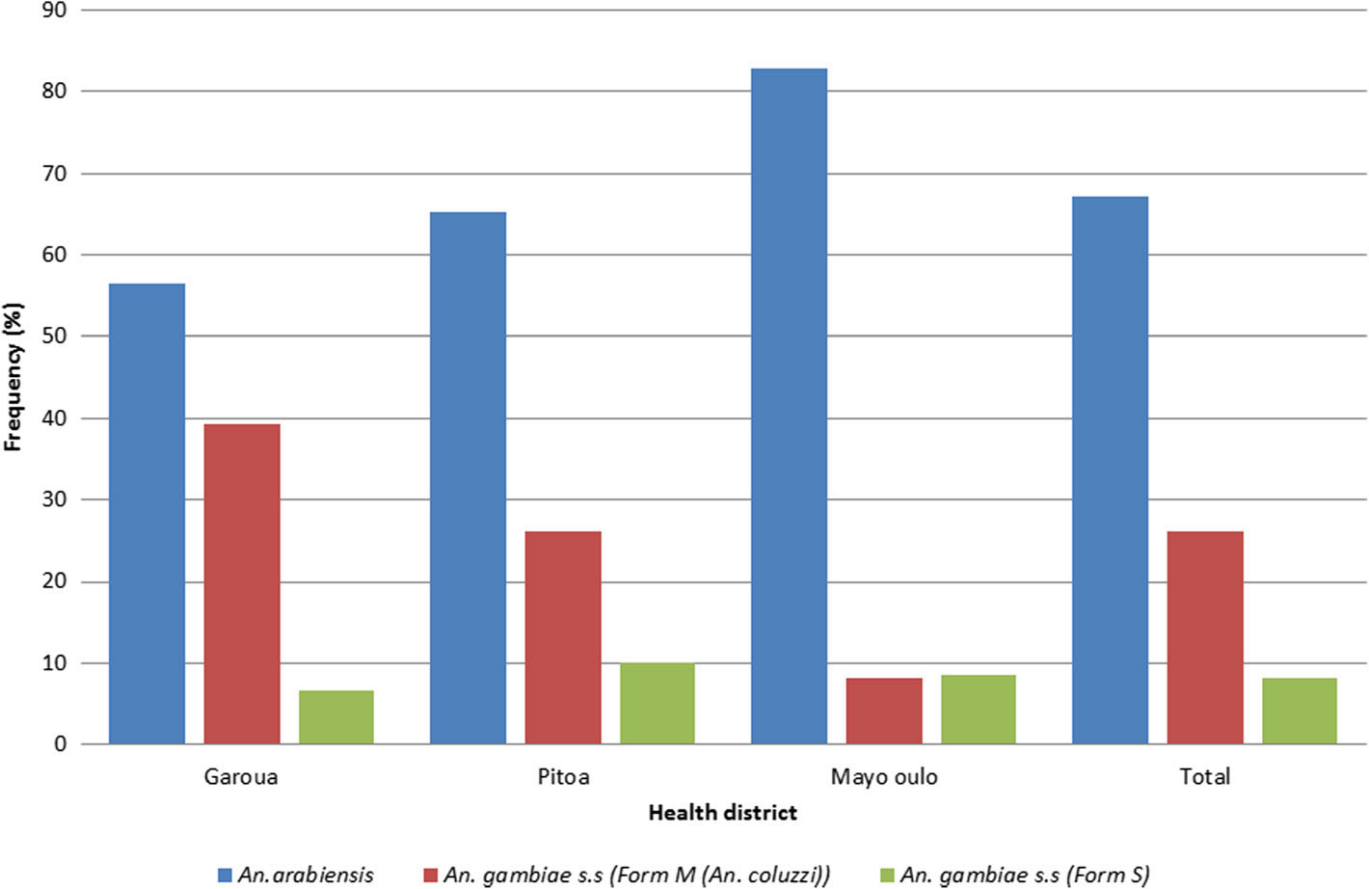
Distribution of members of *Anopheles gambiae* complex in the three study health districts

A total of 1,002 *An. gambiae* (*s.l.*) collected by HLC between 2011 and 2014 were identified by molecular methods. There were 664 (66.27%) *An. arabiensis*, 258 (25.75%) *An. coluzzii* and 80 (7.98%) *An. gambiae* (*s.s.*). Stratifying by health district, there were a total of 314 *Anopheles gambiae* (*s.l.*) tested in Mayo Oulo amongst which there were 83.12, 8.28 and 8.60% of *An. arabiensis*, *An. coluzzii* and *An. gambiae* (*s.s.*), respectively. In Garoua, 426 were analysed comprising 55.28, 38.30, and 6.42% of *An. arabiensis*, *An. coluzzii* and *An. gambiae* (*s.s.*), respectively. Meanwhile in Pitoa, out of 252 *An. gambiae* (*s.l.*) identified, there were 64.29% *An. arabiensis*, 25.79% *An. coluzzii* and 9.92% *An. gambiae* (*s.s.*). Thus, *Anopheles arabiensis* was the most abundant of the *An. gambiae* (*s.l.*) siblings collected by HLC in all three health districts, followed by *An. coluzzii* (Fig. 2). Based on analyses of anophelines collected by PSC, a total of 888 anopheles species comprising 472 (53.15%) *An. gambiae* (*s.l.*)﻿, 364 (40.99%) *An. funestus* and 52 (5.86%) *An. rufipes* were collected. Amongst the members of the *An. gambiae* (*s.l.*), *An. arabiensis* (77.97%) was the most abundant, followed by *An. coluzzii* (16.95%) and *An. gambiae* (5.08%).

### Night biting rate and biting cycles

**Fig. 3:**
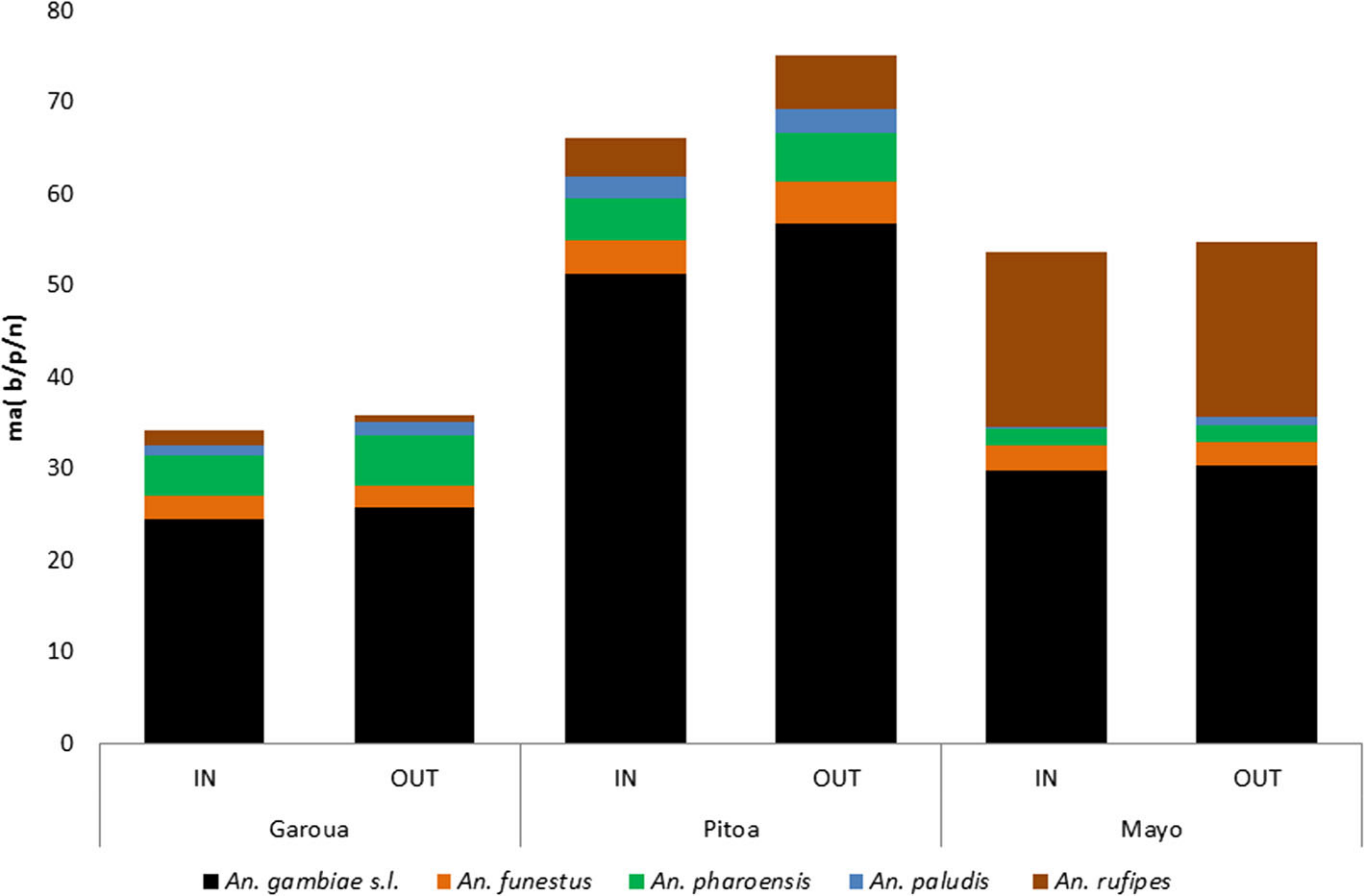
Biting habits of the main *Anopheles* species in the three study health districts. *Abbreviations*: IN, indoor; OUT, outdoor; ma, man biting rate

The human-biting rates varied by species and health district. While peak biting rates for *An. gambiae* (*s.l.*) were recorded in Garoua and Pitoa health districts, in Mayo Oulo *An. rufipes* had the highest biting rates in 2012 and 2013 at 11.083 b/p/n and 7.852 b/p/n respectively (Table 2). The human-biting rate of *An. funestus, An. pharoensis* and *An. paludis* were higher in Pitoa HD compared to Garoua and Mayo Oulo HD. There was no preference to the place of bite for the main *Anopheles* species as they could bite indoor as well as outdoor (Fig. 3).

**Table 2:** Average man biting rate (number of bites per person per night) for *Anopheles* species in the study sites from 2011 to 2014

*Anopheles* species or complex	Garoua				Pitoa				Mayo Oulo			
	2011	2012	2013	2014	2011	2012	2013	2014	2011	2012	2013	2014
*An. gambiae* (*s.l.*)	20.14	8.90	17.12	4.21	39.74	24.26	22.11	21.88	39.36	3.47	6.24	11.16
*An. funestus*	1.14	0.77	1.92	1.02	4.47	2.01	0.94	0.90	1.89	1.28	1.04	1.00
*An. pharoensis*	6.23	0.12	2.72	0.83	4.22	0.65	2.88	2.05	2.06	0.08	1.06	0.67
*An. paludis*	0.03	1.19	1.12	0.32	1.81	1.94	1.28	0.04	0.03	0.11	0.48	0.33
*An. rufipes*	0.66	0.66	0.44	0.54	5.49	3.57	0.47	0.51	8.42	11.08	7.85	10.83

**Fig. 4:**
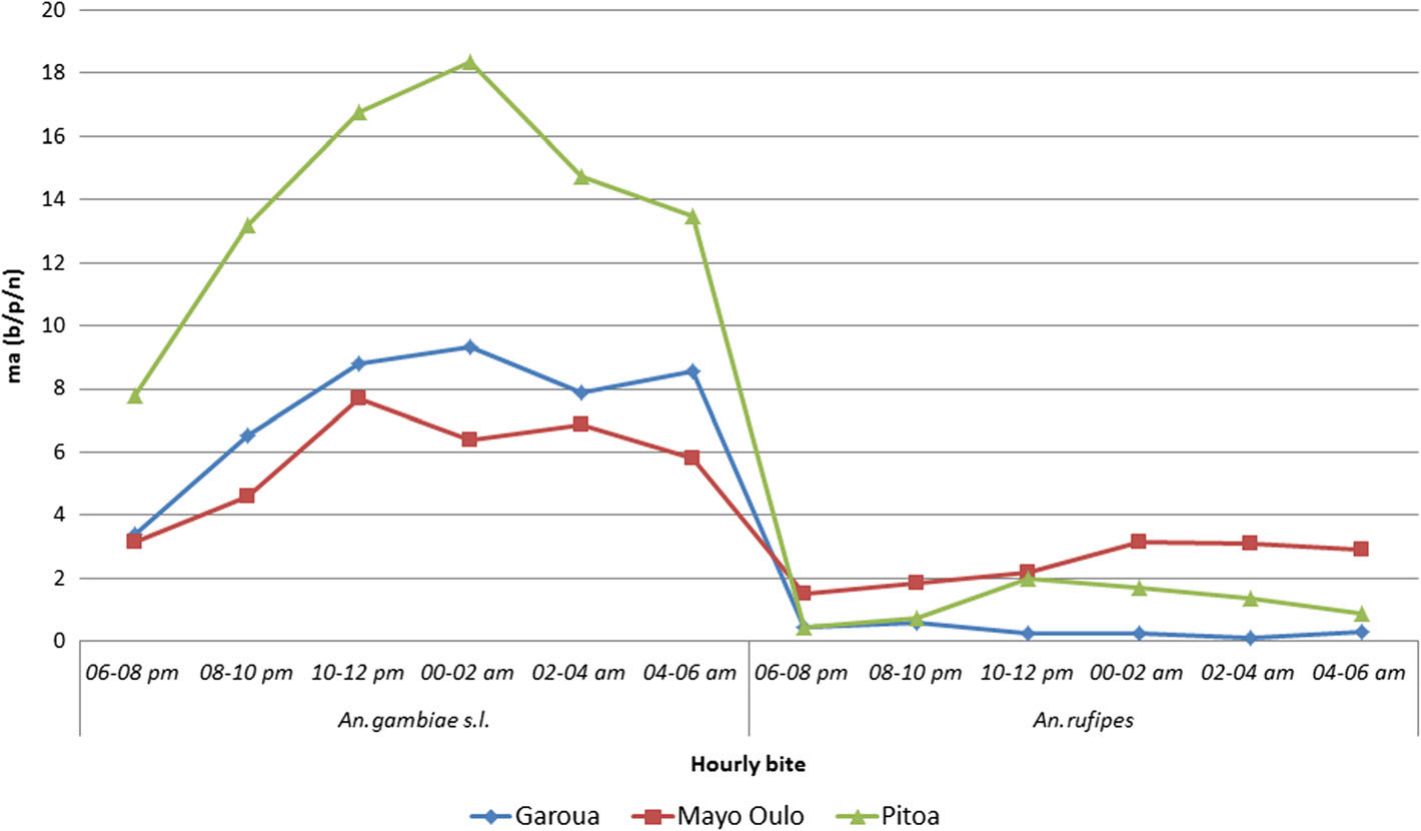
Hourly biting activity of *Anopheles* vector species in three health districts. *Abbreviation*: ma, man biting rate

Comparative analysis of the biting trends by hour of the night between *An. rufipes* and the major vector *An. gambiae* (*s.l.*) showed great variation in the peak aggressivitiy between the health districts (Fig. 4). While *Anopheles gambiae* (*s.l.*) was as most aggressive between 24:00 and 2:00 h in Pitoa and Garoua, in Mayo Oulo its peak activity was earlier, between 22:00 and 24:00 h. On the other hand, *An. rufipes* was most aggressive between 20:00 and 24:00 h in Garoua, 22:00 and 24:00 h in Pitoa, and in the mountainous Mayo Oulo, it was most active between 24:00 and 06:00 h (Fig. 4).

### Parity rates

Out of a total of 10,225 ovaries of *Anopheles* species dissected, 4,438 were parous, giving an overall parity rate of 43.40%. The parity rate was higher for *An. gambiae* (*s.l.*), 46.92% (45.77–48.08), compared to *An. funestus*, *An. rufipes*, *An. pharoensis* and *An. paludis* with parity rates of 37.92, 36.94, 31.12 and 27.67%, respectively (Table 3). With regard to the health district, although generally the highest parity rates were observed in Pitoa, the difference in parity rate between HDs and anopheline species was not significant (*χ* ^2^ = 6.62, *df* = 8, *P* = 0.58). However, there was a significant difference in *An. gambiae* (*s.l.*) parity rates between the health districts (*χ* ^2^ = 8.42, *df* = 2, *P* = 0.0145), which could directly impact on their relative contribution to transmission in the HDs.

**Table 3:** Parity rates of *Anopheles* vectors of malaria in the three study health districts

Health district		*An. gambiae* (*s.l.*)	*An. funestus*	*An. pharoensis*	*An. paludis*	*An. rufipes*
Garoua	No. dissected (No. parous)	2,119 (765)	449 (177)	276 (68)	69 (18)	80 (25)
	Parous rate (%) (95% CI)	36.10 (34.08–38.17)	39.42 (35.01–44.01)	24.64 (19.93–30.05)	26.09 (17.19–37.51)	31.25 (22.15–42.07)
Pitoa	No. dissected (No. parous)	3,856 (2,207)	367 (132)	342 (124)	172 (49)	178 (84)
	Parous rate (%) (95% CI)	57.24 (55.67–58.79)	35.97 (31.23–41.00)	36.26 (31.34–41.48)	28.49 (22.27–35.65)	47.19 (39.99–54.51)
Mayo Oulo	No. dissected (No. parous)	1,184 (387)	136 (52)	60 (19)	12 (3)	925 (328)
	Parous rate (%) (95% CI)	32.69 (30.07–35.41)	38.24 (30.50–46.62)	31.67 (21.31–44.23)	25.00 (8.89–53.23)	35.46 (32.44–38.60)
Total	No. dissected (No. parous)	7,159 (3,359)	952 (361)	678 (211)	253 (70)	1,183 (437)
	Parous rate (%) (95% CI)	46.92 (45.77–48.08)	37.92 (34.89–41.04)	31.12 (27.75–34.70)	27.67 (22.52–33.48)	36.94 (34.24–39.73)

*Note*: The difference in parity rate between health districts and anopheline species was not significant (*χ* ^2^ = 6.62, *df* = 8, *P* = 0.58)

*Abbreviation*: *CI* confidence interval

### Human blood index (HBI)

**Fig. 5:**
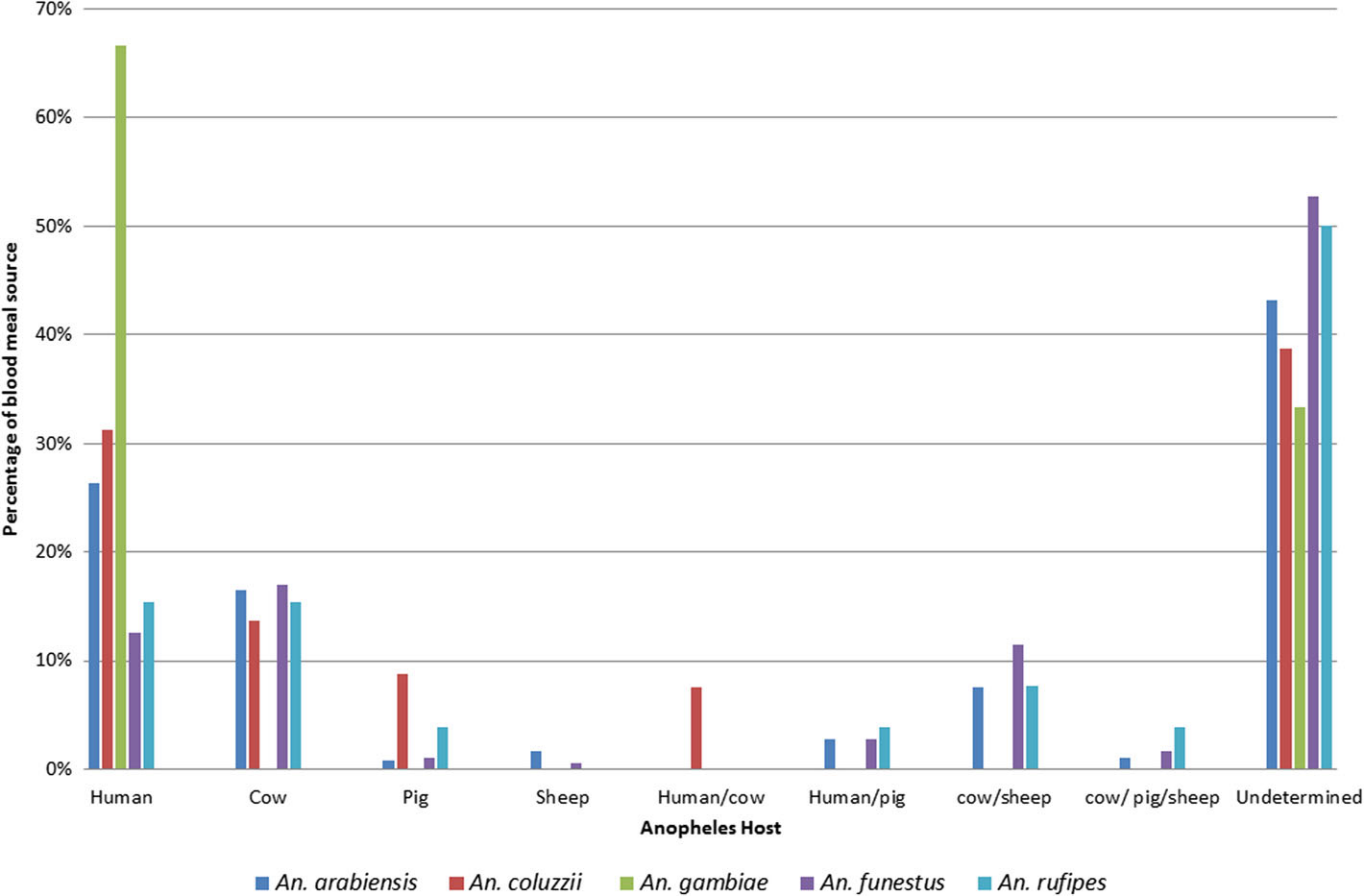
Percentage of blood meal origin of the main *Anopheles* species in the three health districts

In total, the blood meal source of 472 (53.15%) out of the 888 blood-fed *Anopheles s*pecies tested were confirmed. Of these, 21.62% were solely of human origin, 15.99% were from cow and 1.80% from pig. However, there were cases of mixed blood from varied sources such as cow and sheep (8.33%), human and pig (2.48%), and cow, pig and sheep (1.35%). Amongst the members of *An. gambiae* complex, *Anopheles gambiae* was observed to feed essentially on human blood, while *An. arabiensis* and *An. coluzzii,* alongside other species like *An. funestus* and *An. rufipes* fed both on humans and a wide range of domestic animals. About 47% of the source of blood meal from fed *Anopheles* species was not identified, probably due to the lack of monoclonal antibodies to test for blood meals from several other animal species commonly found in those localities (horse, goat, donkey and dog) (Fig. 5). Generally, *Anopheles gambiae* was the most anthropohilic of the *Anopheles* species tested. Also, it had the highest human blood index (HBI) (66.67%) amongst the members of the *An. gambiae* complex (*χ* ^2^ = 23.36, *df* = 2, *P* < 0.05) followed by *An. coluzzii* (31.25%) and *An. arabiensis* (26.36%). The HBI for *An. rufipes* was 15.38% while *An. funestus* had the least (12.64%).

### Infectivity and entomological inoculation rate (EIR)

**Fig. 6:**
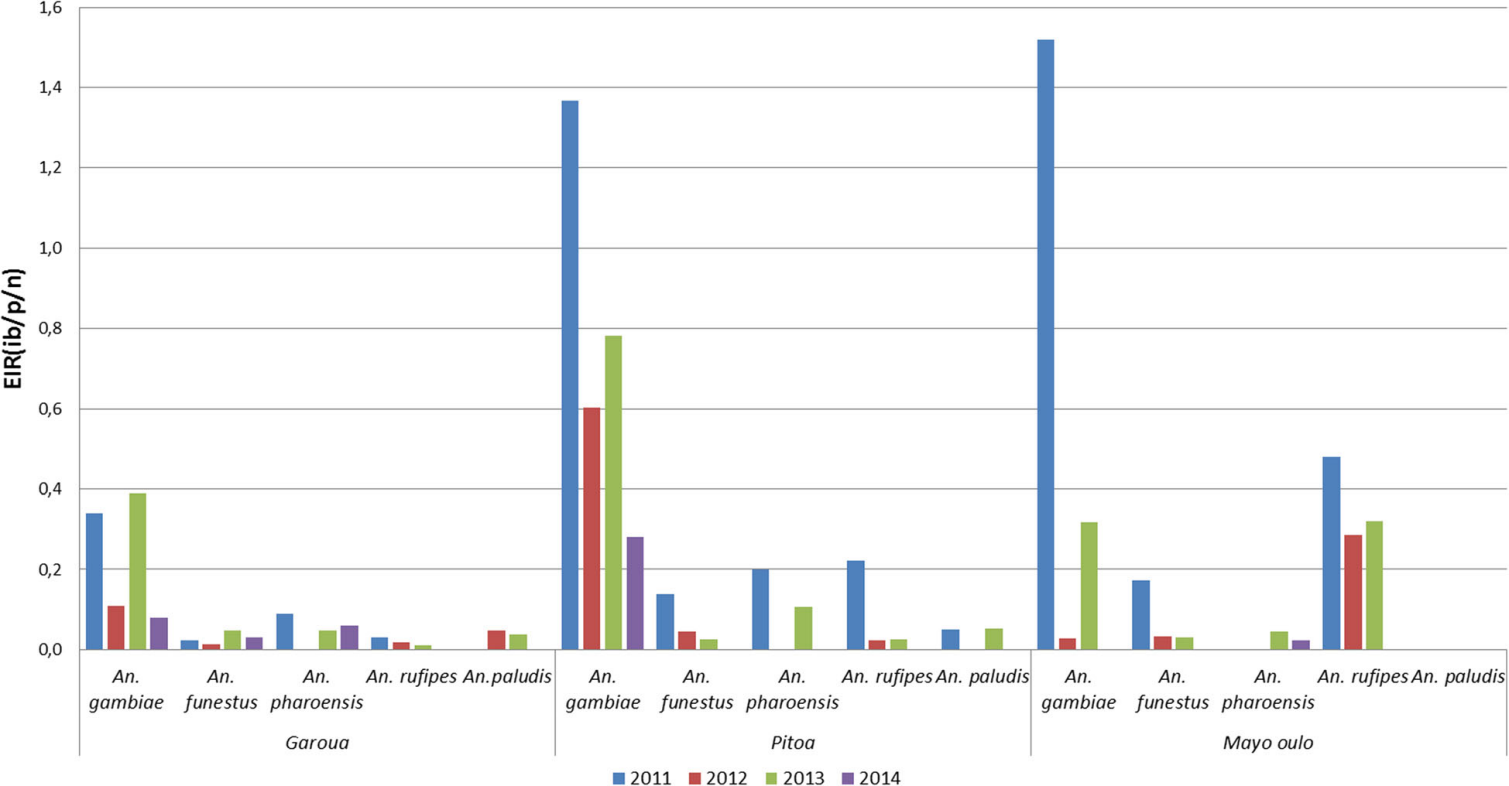
Night entomological inoculation rates of *Anopheles* species by year and health district. *Abbreviation*: ib/p/n, infective bites per person per night

Details of the EIR by vector species and health district are presented in Tables 4 and 5. Overall, the CSP rates varied from 0.8 to 9.1% (Table 4). Irrespective of the year and health district, data indicated that *An. gambiae * (*s.l.*) was responsible for most of the *Plasmodium* transmission. In Mayo Oulo where the highest number of *An. rufipes* was collected, this species accounted for EIR values ranging from 0.286 to 0.481 infective bites per person per night (ib/p/n) in 2012 and 2011, respectively. However, the highest EIR for *An. gambiae* (*s.l.*) (1.52 ib/p/n) was obtained in 2011 (Fig. 6). The highest EIR for *An. funestus* and *An. pharoensis* were 0.032 ib/p/n in 2012 and 0.044 ib/p/n 2013, respectively. In Pitoa health district, *An. gambiae* (*s.l.*) was also responsible for most of the transmission having EIR rates between 0.280 ib/p/n in 2014 and 1.368 ib/p/n in 2011. While the EIR for *Anopheles rufipes* ranged from 0.022 to 0.222 ib/p/n, peak values for *An. funestus*, *An. pharoensis* and *An. paludi*s were 0.139 ib/p/n, 0.200 ib/p/n and 0.052 ib/p/n, respectively during the 4 years. The lowest EIR values were obtained in Garoua HD. Here, the EIR for *An. gambiae* (*s.l.*) varied between 0.079 ib/p/n in 2014 and 0.340 ib/p/n in 2011. For *An. pharoensis* the highest EIR was 0.090 ib/p/n in 2011.

**Table 4:** Summary of overall entomological data

Health district	Anopheline species	2011				2012				2013				2014			
		CSP+ (*n*)	IR	ma	EIR	CSP+ (*n*)	IR	ma	EIR	CSP+ (*n*)	IR	ma	EIR	CSP+ (*n*)	IR	ma	EIR
Garoua	*An. gambiae*	15 (888)	0.017	20.137	0.340	8 (661)	0.012	8.902	0.108	32 (1,408)	0.023	17.118	0.389	6 (318)	0.019	4.206	0.079
	*An. funestus*	1 (49)	0.020	1.137	0.023	1 (56)	0.018	0.775	0.014	4 (158)	0.025	1.922	0.049	2 (70)	0.029	1.020	0.029
	*An. pharoensis*	5 (344)	0.015	6.225	0.090	0 (12)	0	0.118	0	4 (230)	0.017	2.716	0.047	1 (14)	0.071	0.833	0.060
	*An. rufipes*	1 (21)	0.048	0.657	0.031	1 (39)	0.026	0.657	0.017	1 (43)	0.023	0.441	0.010	0 (0)	–	0.539	–
	*An. paludis*	0 (0)	0	0.029	–	2 (51)	0.039	1.186	0.047	3 (88)	0.034	1.118	0.038	0 (7)	0	0.324	0
Pitoa	*An. gambiae*	39 (1,133)	0.034	39.736	1.368	26 (1,045)	0.025	24.264	0.604	14 (396)	0.035	22.111	0.782	11 (859)	0.013	21.880	0.280
	*An. funestus*	5 (161)	0.031	4.472	0.139	2 (88)	0.023	2.014	0.046	1 (36)	0.028	0.944	0.026	0 (31)	0	0.9	0
	*An. pharoensis*	8 (169)	0.047	4.222	0.200	0 (31)	0	0.653	0	2 (54)	0.037	2.875	0.106	0 (10)	0	2.05	0
	*An. rufipes*	4 (99)	0.040	5.486	0.222	1 (164)	0.006	3.569	0.022	1 (19)	0.053	0.472	0.025	0 (14)	0	0.51	0
	*An. paludis*	2 (74)	0.027	1.806	0.049	0 (60)	0	1.944	0	2 (49)	0.041	1.278	0.052	0 (0)	–	0.04	–
Mayo Oulo	*An. gambiae*	19 (492)	0.039	39.361	1.520	1 (122)	0.008	3.472	0.028	8 (157)	0.051	6.241	0.318	0 (24)	0	11.163	0
	*An. funestus*	2 (22)	0.091	1.889	0.172	1 (40)	0.025	1.278	0.032	1 (33)	0.030	1.037	0.031	0 (0)	–	1	–
	*An. pharoensis*	0 (36)	0	2.056	–	0 (2)	0	0.083	0	1 (24)	0.042	1.056	0.044	1 (29)	0.034	0.667	0.023
	*An. rufipes*	2 (35)	0.057	8.417	0.481	4 (155)	0.026	11.083	0.286	2 (49)	0.041	7.852	0.320	0 (0)	–	10.833	–
	*An. paludis*	0 (0)	0	0.028	–	0 (3)	0	0.111	0	0 (13)	0	0.481	0	0 (0)	–	0.333	–

*Abbreviations*: *CSP+* number of mosquitoes positive to CSP, *CSP* circumsporozoite protein, *EIR*entomological inoculation rate (infectious bites/person/night, *IR* infection rate, *ma* man biting rate (bites/person/night), *n* number of mosquitoes examined by CSP ELISA

**Table 5:** Implication of members of *Anopheles gambiae* complex in malaria transmission

Health district	Anopheline species	2011				2012				2013				2014			
		CSP+ (*n*)	ma	IR	EIR	CSP+ (*n*)	ma	IR	EIR	CSP+ (*n*)	ma	IR	EIR	CSP+ (*n*)	ma	IR	EIR
Garoua	*An. arabiensis*	1 (78)	0.76	0.01	0.01	1 (31)	0.3	0.03	0.01	3 (75)	0.74	0.04	0.029	1 (7)	0.07	0.14	0.0098
	*An. coluzzii*	1 (49)	0.48	0.02	0.01	0 (23)	0.23	0	0	1 (42)	0.41	0.02	0.01	0 (18)	0.18	0	0
	*An. gambiae*	0 (11)	0.11	0	0	0 (7)	0.07	0	0	1 (2)	0.02	0.5	0.01	0 (4)	0.04	0	0
Pitoa	*An. arabiensis*	2 (113)	1.57	0.02	0.028	1 (67)	0.93	0.01	0.014	1 (86)	1.19	0.01	0.014	1 (71)	0.7	0.01	0.0098
	*An. coluzzii*	1 (67)	0.93	0.01	0.014	1 (33)	0.46	0.03	0.014	0 (15)	0.21	0	0	1 (20)	0.2	0.05	0.0098
	*An. gambiae*	0 (14)	0.19	0	0	0 (17)	0.24	0	0	0 (6)	0.08	0	0	0 (15)	0.15	0	0
Mayo Oulo	*An. arabiensis*	5 (83)	1.54	0.06	0.093	2 (6)	0.11	0.33	0.037	2 (17)	0.31	0.12	0.037	0 (3)	0.06	0	0
	*An. coluzzii*	0 (8)	0.15	0	0	0 (1)	0.02	0	0	0 (3)	0.06	0	0	0 (0)	0	–	–
	*An. gambiae*	1 (5)	0.09	0.2	0.019	0 (1)	0.02	0	0	0 (2)	0.04	0	0	0 (2)	0.04	0	0

*Abbreviations*: *CSP+* number of mosquitoes positive to CSP, *CSP* circumsporozoite protein, *EIR* entomological inoculation rate (infectious bites/person/night, *IR* infection rate, *ma* man biting rate (bites/person/night), *n* number of mosquitoes examined by CSP ELISA

With regards to the members of the *Anopheles gambiae* complex, although in general *An. arabiensis* recorded the highest EIR, with peak values of 0.029 ib/p/n in 2013 in Garoua, 0.028 ib/p/n in 2011 in Pitoa and 0.093 ib/p/n in 2011 in Mayo Oulo, the difference was not significant when compared to *An. colluzii* in Garoua and Pitoa (*χ* ^2^ = 0.014, *df* = 3, *P* > 0.05). However, *An. gambiae* contributed to transmission mainly in 2011 in Mayo Oulo with an EIR of 0.019 ib/p/n and in 2013 in Garoua with an EIR of 0.01 ib/p/n (Table 5).

## Discussion

The identification and incrimination of vectors in malaria transmission as well as their relative contribution to transmission is critical for the implementation of an efficient malaria control strategy. Some control programmes like the Garki project, failed because of the erroneous identification of the vectors involved in malaria transmission, and insecticide resistance in *An. arabiensis* in Nigeria [35, 36]. To date, malariologists have paid little or no attention in most areas to *An. rufipes* as this species has generally exhibited high zoophilic tendencies. Elsewhere, the abundance of this species was found to peak particularly at the end of the rainy season in rice-growing areas, with very short periods of malaria outbreak resulting [17–20]. *Anopheles rufipes* breed commonly in small collections of water such as residual puddles in river beds, hoof prints of cattle, swamps and rice irrigation schemes, with the larvae staying close to the borders and among vegetation. This is important as cattle rearing and rice cultivation and irrigation are common practices in the study sites, which usually experience floods with long retention of surface water during the wet season. As part of a study to determine the impact of insecticide resistance on the effectiveness of vector control tools like the LLIN in the north of Cameroon, the observed unexpectedly high density and anthropophilic behaviour of this species incited our curiosity to investigate its role in *P. falciparum* transmission. For the first time in Cameroon, *An. rufipes* has been identified as an important local vector of *P. falciparum.* A few earlier studies have described this species as weakly anthropophilic [18] and potentially involved in malaria transmission [3, 37, 38]. More recently in Burkina Faso, *Plasmodium* was detected in *An. rufipes* oocyts, further implicating it as a potential vector of human malaria parasites [39]. Thus, *An. rufipes* may be a more important and wider spread vector than previously thought and therefore this is important for malaria elimination strategies.

In Cameroon, five major malaria vectors (*An. gambiae*, *An. funestus*, *An. arabiensis*, *An. nili* and *An. moucheti*) have been described [10] with nine secondary vectors of local importance (*An. paludis*, *An. carnevalei*, *An. coustani*, *An. marshallii*, *An. ziemanni*, *An. pharoensis*, *An. hancocki*, *An. wellcomei* and *An. ovengensis*). *Anopheles rufipes* is now added to this plethora of vectors, further complicating the malaria vectorial system in the country. In the past, the role of this species in malaria transmission was largely neglected due its strong zoophilic tendency which leads to claims of its lack of importance. Moreover, *An. rufipes* had high parous rate wherever it was collected, suggesting the propensity to survive longer and being able to maintain and even extend *P. falciparum* transmission beyond the normal duration.

The vast plethora of *Anopheles* species observed in this study could be linked to the diversity of existing biotopes that enhance the proliferation of such species within the health districts. *Anopheles arabiensis*, *An. coluzzii* and *An. gambiae* (*s.s.*) were  the *An. gambiae* (*s.l.*) siblings found with *An. arabiensis* being the most frequently encountered species. The relative abundance of *An. gambiae* (*s.s.*) and *An. arabiensis* varied across the years and by health districts with twice as many *An. arabiensis.* This is unlike observations by Robert et al. [40] in southern Burkina where the density of *An. gambiae* (*s.s.*) was nine-fold greater than that of *An. arabiensis* among residual endophilic mosquitoes. This difference however, might be due to differences in the microenvironments and the methods of collection. Both species are typical malaria vectors of the sahelian zone [41], with their densities peaking especially during the rainy season. As observed with *An. rufipes* in this study*,* species like *An. pharoensis*, *An. funestus* and *An. coustani* have been shown to contribute significantly to malaria transmission [23]. For *An. funestus*, the density remained relatively constant irrespective of the season [42]. Akin to studies conducted in Burkina Faso, *An. rufipes* constituted a significant proportion of the anophelines collected in this study [39]. This however differs markedly with observations from the HLC collections in the coastal areas and northern regions of Cameroon [21, 22] and Chad [43], where *An. rufipes* density was low. The vast number of prolific breeding sites (rice fields, river banks and pools) during the study periods may explain the high density of *An. rufipes* [19, 20].

With regard to the biting rates of the anophelines, except in Mayo Oulo where the highest *An. rufipes* densities were recorded in 2012 and 2013, *An. gambiae* (*s.l.*) generally had the highest biting rates throughout the study (Table 2). Analyses of the blood meal sources among the members of the *An. gambiae* complex revealed *An. gambiae* despite its low density compared to *An. coluzzii* and *An. arabiensis*, to be the most anthropophagic. Amongst the vectors for which the blood meal source was confirmed, they were observed to feed mainly on cow, which is the most widely domesticated animal in this part of the country. *Anopheles rufipes* was observed to feed on both humans and animals confirming the species opportunistic behaviour or may be simply due to the imposing environmental conditions. Livestock rearing is a common practice with animals like dog, chicken, horse, goat and donkey in addition to those analyzed. Unlike previous reports on *An. funestus* being the most anthropophagic *Anopheles* species [33, 44–47] its human blood index in this study was very low. This may be due to the presence of a variety of domestic animals that provide an alternative source for blood-feeding or due to the occurrence of other members of the *An. funestus* group of species. In fact, *Anopheles funestus* (*s.l.*) comprises  at least nine species that are morphologically identical [3]: *An. funestus* (*s.s.*), *An. vaneedeni* Gillies & Coetzee, *An. parensis* Gillies, *An. aruni* Sobti, *An. confusus* Evans & Leeson, *An. rivulorum* Leeson, *An. fuscivenosus* Leeson, *An. leesoni* Evans and *An. brucei* Service. All except *An. funestus* (*s.s.*) and to some extent *An. rivulorum* are purported to be exclusively zoophilic and non-vectors [48]. Despite the fact that many authors reported a high infection rate of *An. funestus* during dry season [3, 49], Antonio-Nkondjio et al. [22] observed a pronounced anthropohilic behaviour of *An. funestus* during the wet season while this species was exclusively zoophilic during the dry season. Other earlier studies [50, 51] also reported besides *An. funestus* (*s.s.*) the presence of *Anopheles leesoni* Evans and *Anopheles rivulorum*-like in tributaries of the Benoue River in the northern region of Cameroon. Based on these observations and the current HBI results, there is the likelihood of the existence of some zoophilic members of *An. funestus* group in this area. Therefore, it would be important that subsequent studies in this area should consider the identification of the members of the *An. funestus* group of species and their contribution to human malaria transmission.

On a general note, in addition to the *Anopheles* species displaying endophagic and exophagic tendencies, the parity rates were observably high in all HDs. Thus, despite the high LLIN coverage in the study localities [52], the malaria vectors survive long enough to sustain malaria transmission locally.

The infection rates varied enormously between the health districts. Overall, seven *Anopheles* species (*An. arabiensis*, *An. coluzzii*, *An. gambiae*, *An. funestus*, *An. pharoensis*, *An. rufipes* and *An. paludis*) were found infected with *P. falciparum*. Even though the CSP rates were higher than those obtained elsewhere under similar eco-epidemiological conditions [22]*,* measures were taken to minimize false positive CS ELISA in these areas with an extensive animal reservoir flow for blood-feeding. Thus, only high absorbance readings (mean plus three standard deviations of negative controls) were considered. Nevertheless, this CSP-ELISA may overestimate the infection rate and all positive samples need to be confirmed with PCR [53]. Relatively low malaria transmission intensity was observed throughout the years in all health districts. The observed EIR estimate of 0.014 to 1.221 infective bites/person/night was consistent with other records in the sahelian zone [25]. However, the transmission intensity during the study was pronounced in the irrigated rice paddies of Pitoa, which is not uncommon to other such irrigation schemes that generate a variety of entomological and epidemiological situations with permanent water bodies that are prolific for *Anopheles* breeding and consequently increasing the risk of malaria in surrounding population [54].

## Conclusions

The study highlights the epidemiological role of *An. rufipes* alongside the members of the *Anopheles gambiae* complex, and several other sympatric species in human malaria transmission during the wet season in northern Cameroon. Thus, for the first time in Cameroon, the role of *An. rufipes* as an important local malaria vector has been made evident. The study also portrays the need to carry out further studies to document the implication of this species and other presumably non-malaria vectors in human malaria transmission in other parts and to review and update the malaria entomological profile in the country and the Afrotropical region.

## Abbreviations

CSP: Circumsporozoite protein
EIR: Entomological inoculation rates
ELISA: Enzyme-linked-immunosorbent assay
HD: Health district
HLC: Human landing catch
LLINs: Long-lasting insecticide-treated nets
PSC: Pyrethrum spray collection
